# The experience of patients undergoing aseptic, elective revision knee joint replacement surgery: a qualitative study

**DOI:** 10.1186/s12891-024-07778-3

**Published:** 2024-08-29

**Authors:** Philippa J. A. Nicolson, Francine Toye, Shiraz A. Sabah, Andrew J. Price, Abtin Alvand, Karen Barker

**Affiliations:** 1 Physiotherapy Research Unit, Oxford University Hospitals NHS Foundation Trust Nuffield Orthopaedic Centre, Oxford, OX3 7HE UK; 2 Nuffield Department of Orthopaedics, Rheumatology and Musculoskeletal Sciences (NDORMS), University of Oxford, Oxford, UK; 3 Oxford University Hospitals NHS Foundation Trust Nuffield Orthopaedic Centre, Oxford, UK

**Keywords:** Revision knee replacement, Qualitative research, Patient experience

## Abstract

**Background:**

Around 6,000 revision knee replacement procedures are performed in the United Kingdom each year. Three-quarters of procedures are for aseptic, elective reasons, such as progressive osteoarthritis, prosthesis loosening/wear, or instability. Our understanding of how we can best support these patients undergoing revision knee replacement procedures is limited. This study aimed to explore patients’ experiences of having a problematic knee replacement and the impact of undergoing knee revision surgery for aseptic, elective reasons.

**Methods:**

Qualitative semi structured interviews with 15 patients (8 women, 7 men; mean age 70 years: range 54–81) who had undergone revision knee surgery for a range of aseptic, elective indications in the last 12 months at an NHS Major Revision Knee Centre. Interviews were audio-recorded, transcribed, de-identified and analysed using reflexive thematic analysis.

**Results:**

We developed six themes: Soldiering on; The challenge of navigating the health system; I am the expert in my own knee; Shift in what I expected from surgery; I am not the person I used to be; Lingering uncertainty.

**Conclusions:**

Living with a problematic knee replacement and undergoing knee revision surgery has significant impact on all aspects of patients’ lives. Our findings highlight the need for patients with problematic knee replacements to be supported to access care and assessment, and for long-term psychological and rehabilitation support before and after revision surgery.

**Supplementary Information:**

The online version contains supplementary material available at 10.1186/s12891-024-07778-3.

## Background

More than 100,000 primary knee replacements were performed in the United Kingdom (UK) in 2022, and this number is expected to rise in the coming years [1]. The increasing demand for primary knee replacement has resulted in a rise in revision knee surgery, with over 5,700 revision knee replacements performed in the UK in 2022 [1, 2].

A revision knee replacement is a surgical procedure to replace, modify, or wash out a joint replacement that is no longer functioning correctly [1]. Revision knee replacements are both expensive and high-risk [3]. The majority (> 70%) of revision knee replacements are undertaken for aseptic, elective reasons such as instability, progressive osteoarthritis, stiffness, or unexplained pain. The goal of an aseptic, elective revision knee replacement is to reduce pain, and improve function and quality of life. A smaller proportion of revisions are undertaken for urgent and non-discretionary reasons, such as infection or fracture, where the main goal of surgery may differ. Clinical outcomes following revision knee joint replacement are highly variable and often inferior to those after primary knee replacement [4]. Given the significant cost of revision knee replacement surgery, the health economic costs of these variable outcomes are also significant [5].

While extensive research has explored the experience of patients [6–10], and interventions to support patients before and after primary knee joint replacement [11], research focused on revision knee joint replacement is more limited. The James Lind Alliance has identified two of the top ten research priorities for problematic knee arthroplasty [12]: ‘What is the psychological impact of a problematic knee arthroplasty and what support do people need before, during and after revision knee surgery?’ and ‘What can be done after and/or before revision knee surgery to optimize the result?’. It is essential to understand patients’ experiences and the impact of revision knee replacement to inform future design of rehabilitation interventions to improve patient outcomes.

A small number of qualitative studies have examined the experience of patients undergoing revision hip or knee joint replacement specifically for prosthetic joint infection [13, 14], but to our knowledge, no studies have yet explored the experience of the much larger group of patients undergoing aseptic, elective revision knee replacement surgery.

The aim of this study was to gain a deeper understanding of patients’ experiences of having a problematic knee replacement and the impact of undergoing revision knee joint replacement for aseptic, elective reasons.

## Methods

### Design

Qualitative study using semi structured interviews. We took a phenomenological approach to explore patients’ subjective perspectives about their lived experience of having a problematic knee replacement and the impact of undergoing revision knee joint replacement for aseptic, elective reasons. This research was approved by the Wales Research Ethics Committee 6 under (reference 22/WA/0265).

### Sampling, recruitment and consent

We recruited study participants from follow-up outpatient orthopaedic clinics at one National Health Service (NHS) Major Revision Knee Centre in England between 1 January 2023 and 31 May 2023.

Inclusion criteria were: adults, male or female, aged 18 years or above; revision knee replacement in the last 12 months due to an aseptic, elective indication (e.g. loosening, component wear, stiffness, unexplained pain). Exclusion criteria were: Unable or unwilling to give informed consent; revision or re-revision total knee replacement due to sarcoma, infection, or fracture.

The sample was purposive, guided by the concept of information power as proposed by Malterud [15]. The exact sample size was not predetermined, instead recruitment ceased when sufficient information power was reached. Our study had a relatively broad aim, our sample was specific, and we had not identified a theoretical framework a priori. The quality of dialogue was strong, and analysis was exploratory and cross-case. Referring to the research team’s experience we estimated that a purposive sample of 12 to 20 participants would provide sufficient information power.

Members of the clinical care team identified potentially eligible participants who met the inclusion criteria from revision knee replacement follow up clinic lists. Members of the clinical care team sent a study invitation pack which included an invitation letter, the patient information sheet. Potential participants were asked to return the reply slip to the research team if they were interested in taking part. The researcher (PN) contacted potential participants via telephone to confirm eligibility, explain the interview study, and answer any questions. An interview was scheduled if the potential participant was happy to proceed. We offered participants the choice of being interviewed via a telephone call, via video conferencing or in-person.

We obtained informed consent verbally from participants before starting the interview as all participants chose to be interviewed via telephone. The researcher read through the consent statements and completed the paper consent form on behalf of the participant, then signed the form, scanned, and posted a copy to the participant.

### Data collection

We developed a semi structured interview guide and open-ended interview with the research team, which included an anthropologist, two clinical academic physiotherapists, three clinical academic orthopaedic surgeons and two patient partners who had undergone revision knee surgery themselves (Additional File 1). One researcher (PN), a clinical academic physiotherapist, experienced in qualitative research, who was not known to the participants in a clinical capacity conducted all interviews. Interviews lasted between 20 min and 50 min and were digitally recorded and transcribed verbatim, with permission.

### Data analysis

We uploaded interview transcripts to NVIVO software to assist our organisation of analysis (V10.0, QSR International Pty Ltd., Doncaster, Victoria, Australia). We used the six stages of reflexive thematic analysis to develop themes that were identified across participants: (a) familiarisation; (b) coding (distilling narrative into meaning units); (c) generate initial themes; (d) develop and review themes; (e) refining and naming themes: (f) writing up [16]. Two authors (KB, PN (both clinical academic physiotherapists)) independently read and coded each interview transcript. Themes were developed, discussed, and reviewed by three researchers (PN, KB and FT).

## Results

We interviewed 15 participants who had undergone revision knee replacement surgery in the previous 12 months. Interviews were conducted between January and May 2023. Characteristics of the participants are summarised in Table 1. Participants were a median of 8 months post-surgery (range: 6 to 10 months). The median age of participants was 68 years (range: 54 to 81 years), and 53% (*n* = 8) were females. Participants were from a range of areas of social advantage as measured by the Index of Multiple Deprivation (IMD): median: 4 (range: 1 (1 participant) to 5 (3 participants)); 1 being most deprived and 5 being least deprived (Table 1). Revision knee replacement surgery was most commonly single stage (*n* = 13, 87%), and was undertaken for osteoarthritis progression (*n* = 6, 40%), prosthesis loosening or wear (*n* = 5, 33%) or instability (*n* = 2, 13%). Participants were a median of 9.5 years post primary knee replacement surgery (range from 6 months (1 participant) to more than 15 years (4 participants)). In the time since their primary knee replacement two participants had undergone four or more additional surgical procedures on their knee.

**Table 1:** Characteristics of participants (*n* = 15)

Pseudonym	Gender	Age	Work status	IMD quintile*	Revision procedure	Indication	Months since revision surgery	Years since primary knee replacement	Number of operations since primary joint replacement
Gwen	Female	76	Retired	4	Single Stage	Instability	8	13	1
Rory	Male	63	Working fulltime	3	Two Stage	Loosening / wear	7	2	4
Anne	Female	68	Retired	2	Single Stage	Loosening / wear	8	5	0
Neville	Male	67	Retired	4	Single Stage	Loosening / wear	6	9	2
Bob	Male	76	Retired	5	Single Stage	Instability	8	1	1
John	Male	81	Retired	5	Single Stage	OA Progression	8	5	0
Elaine	Female	75	Retired	5	Single Stage	OA Progression	8	1	0
Helen	Female	81	Retired	4	Single Stage	Loosening / wear	8	0.5	0
Mark	Male	54	Unable to work	1	Single Stage	Loosening / wear	7	17	5
Dianne	Female	66	Working part-time	4	Single Stage	Fractured bearing	10	10	0
Frank	Male	72	Retired	4	Single Stage	Dislocated bearing	8	1	1
Gayle	Female	67	Working part-time	5	Single Stage	OA Progression	6	16	0
June	Female	76	Retired	4	Single Stage	OA Progression	6	18	1
Trevor	Male	62	Working part-time	2	Single Stage	OA Progression	6	17	0
Ruth	Female	68	Working part-time	2	Single Stage	OA Progression	10	2	1

* Index of Multiple Deprivation presented by quintiles: quintile 1 being most deprived and quintile 5 being least deprived

We report six themes, illustrated with verbatim narrative. Themes, subthemes and examples of codes are included in Additional File 2. Our findings demonstrate the over-arching sense from interviewees of “soldiering on”, while managing the challenges of navigating the health system, the struggle to be recognised as the expert in their own knee, shifts in what they expect from surgery and feeling that they are now no longer the person they used to be.

### Soldiering on

This theme describes the need to *“just get on with it” (Anne)* in spite of pain and a sometimes-overwhelming hierarchy of health needs. Participants described that they *“kept going and going because I had to*,* there was no choice” (Gayle)*, and reflected that *“it was hard*,* having to cope every day” (Bob).*

*I don’t let things get on top of me*,* I just carried on as best I could. I think I had to rest a lot*,* which was unusual for me*,* I have always sort of got on with life. Pain-wise*,* I was on so much paracetamol. I had to come off paracetamol because it was affecting my liver. (Helen)*

There was a sense of having to juggle a fluctuating hierarchy of health and social priorities. Several participants felt they “*needed the knee sorted so I could deal with other concerns” (Frank)*. Participants described the challenge of managing multiple health conditions, both their own and those of family members, and having *“to prioritise what is most important to focus your energy on” (June).*

*My husband was diagnosed with memory loss… And I think that impacted on my own reactions*,* because I perhaps should have gone and seen somebody sooner. I suddenly got a lot of pain in that left knee but went on with it because I decided that the time had come for him to be given a chance. And so*,* it wasn’t until very recently that I’ve been and reported that change in the knee. I haven’t thought about it and now I’m wording it*,* it’s come back just how hard it was. (Gwen)*

### The challenge of navigating the health system

This theme describes the challenge of navigating the health care system, exacerbated by having to fight to be heard. Participants described *“repeatedly going back*,* time and time again” (Anne)* but struggling to have their knee symptoms taken seriously by health professionals. Some had to repeatedly request to be referred to other health professionals, or for further investigation of their symptoms. Some felt frustrated and angry that they *“just couldn’t get through to them that the knee wasn’t right” (Neville).*

*I kept telling them… I talked to two GPs*,* went to see a physio and had an x-ray*,* and I kept telling them*,* “This isn’t an injury.” Then I kept trying to get the results for the x-ray and I kept ringing the GP and they said*,* “If it’s okay*,* we won’t get the results*,*” and I said: “Look*,* it’s not getting any better. I’m sure it’s not an injury. Something’s gone wrong with my knee replacement*,*” but I couldn’t get through to them. (Dianne)*

As a result of this battle to be heard and taken seriously, some participants had lost faith in certain health professionals, and others in the health system as a whole.

*When you’re suffering in so much pain*,* and it’s affecting your life*,* and nothing they do makes you feel no better… you start losing faith in your GP*,* and as I said earlier*,* because my GP was off – I kept seeing different doctors. One would say*,* “Have you tried so-and-so?” I’d say “no”*,* and then*,* “We’ll try that”*,* and then naproxen made me bad in my stomach. Then I tried something else that gave me diarrhoea*,* then I tried something else. It’s all crap. They just don’t want to refer you. (Trevor).*

### I am the expert in my own knee

Participants challenges navigating the health care system were further exacerbated by the struggle to balance various clinical opinions with their own health experiences and expertise. Participants emphasised the desire to be ‘in’ the conversation with health professionals, and to be empowered to ask questions and discuss plans.

*It seemed like because I was already a revision returner*,* they put me at the bottom of the list*,* I was a bit upset that he didn’t go into detail as to why this was giving me so much problem. Whether there wasn’t anything on the x-ray*,* I find that … well knowing my body*,* I find that difficult to understand when I’ve been through so much pain*,* that there wasn’t anything that was showing up on the MRI and the x-ray. I’m not a medic*,* but I know my body.” (June)*.

However, participants wanted to be seen as a ‘good patient’ who did not complain or ‘cause trouble’. Some described positive traits to support a moral character as a ‘good patient’ despite the challenge of managing their symptoms, and frustrations at feeling unheard.

*They said*,* come back in a years’ time and I thought*,* oh my god*,* why don’t they believe me. I’m not the sort of person that makes a fuss … I just trust people if they’re a medic and they’re in that position*,* they know what they’re talking about. I’m somebody that hardly ever goes to the doctor*,* I’m not on medication*,* never been on medication…. I’ve been active all my life. So this was a huge blow to me that suddenly my life had stopped. (Anne)*

*There is a lot of scary news on the television and radio about don’t go and trouble your doctor*,* so while I was able to get up and about*,* I didn’t want to bother them. (Helen)*

### Shift in what I expected from surgery

Participants described adjustments in what they hoped to achieve by undergoing revision knee joint replacement. Some felt that they had approached their primary knee joint replacement *“naïvely*,* hoping for complete pain relief” (Helen)*, whereas for subsequent surgeries their expectations and hopes were for *“a degree of pain relief” (Neville)*, or *“for the knee to be tolerable” (Mark).*

*Let’s just take this latest one*,* this left third knee*,* I just hoped for some of the pain relief that I had had over the previous 11 years. Pain relief so that I could get on and do the things I knew I could do okay. (Gwen)*

*I think it’s getting back a degree of normality again. I knew I wasn’t going to be totally pain-free or stiffness-free because I never have been. I wasn’t over-optimistic*,* I wasn’t expecting miracles. (June)*

At times, this shift in expectation meant participants expressed gratitude for even minor improvements, knowing how bad it had been, and how bad it could have been.

*I guess my knee … despite everything I may say*,* they may not have returned to the knees … they’re no longer the knees of a 32-year-old or anything like that*,* but they’re a bloody sight better than the ones I had before I had them changed. (Bob)*

*My knee is still much bigger than the other one*,* but it’s not so painful as it was*,* so I will take that. I have been through too many operations to be worried about what my knees look like. If they couldn’t have done the knee*,* they’d have had to chop my leg off I think*,* above the knee. That would be the only option. So*,* anything to do with being mobile and out of pain was a bonus for me. (Neville)*

### I am not the person I used to be

This theme explored how participants grappled with their own personal and social identity, whilst living with a troublesome knee and after undergoing revision knee replacement. Several participants expressed they found it *“really difficult seeing others have successful knee replacements” (Helen).* Some felt they were no longer able to be the person they were prior to surgery and grieved this loss.

*I’ve got high expectations of life. I’ve obviously had to revise them as I know I’m never going to ski again and I know I’m never going to run again. I was always so active*,* that was a huge part of my life*,* and my family’s life. (Rory)*

Participants also spoke about impacts on social identity, including the experience putting “*a huge strain on family relationships” (Mark)* and social exclusion because of their knee condition: *“people definitely stopped inviting us to things because I was so limited by me knee” (Anne).*

*I felt a bit of a twit saying sorry it’s my knee. I’m so sad that I haven’t been able to do as much as I would normally have done*,* because of the knee – this is the first time that anything has really interrupted the things that I had on my plate. (Gwen)*

Some participants reflected that they were *“disappointed in myself*,* that this all went wrong” (Dianne)* or that they were *“frustrated at my body for letting this happen” (Rory).*

*The regret is I just wish I’d have bit the bullet and gone back to the surgeon and had the knee re-done*,* which it needed doing*,* in the first place. But there you go. I always feel it’s a bit self-inflicted and being quite healthy at the moment and fit*,* it annoys you that you can’t do everything you want to do*,* even now. (Neville)*

### Lingering uncertainty

This theme explores the ongoing uncertainty continuing long after surgery. Participants felt that the experience has “*really shaken my confidence in everything” (Mark)* and that the threat of something going wrong again “*is always in the back of your mind” (Ruth).*

*I still feel frustrated by my knee. I’m seven months since the last operation*,* so*,* you know*,* I’m slightly frustrated that it’s still giving me problems*,* but I’m grateful I’m able to work. I think with everything I have been through that I just want to know that it is improving*,* even very slowly. Because you worry that it might go wrong again*,* that hangs over you for sure. (Rory)*

*I don’t feel completely confident on it. I do worry that it will let me down again. I think the strength’s getting better. I get the impression the bending is never gonna get much more*,* but I can live with that. I do get some hip pain and I think “here we go again”*,* but it seems to settle with rest*,* for now anyway. (Gayle)*

Reflecting on the experience, some participants expressed *“a niggling feeling of how unfair it is that this happened to me” (Mark)*, while others expressed a desire to *“put it behind me and get on with life as much as I can” (Frank)*.

*Nobody has done anything wrong*,* it has just been a bummer really*,* it’s just one of those things. And it happens rarely*,* it’s just I would rather it happen rarely to somebody else rather than me. (Bob)*

## Discussion

We aimed to explore patients’ experiences of living with a problematic knee replacement and undergoing revision knee replacement for aseptic, elective reasons. Our findings highlighted the negative impact of a problematic knee replacement on all aspects of patients’ lives. Participants described the tension between prioritising health needs against social priorities and the fatigue of *soldiering on*. We identified *the challenge of navigating the health system* and the struggle to have their health needs understood and taken seriously by healthcare professionals. There was a lack of continuity of care: multiple appointments for the same complaint, with individuals and systems that often did not communicate with one another.

This study adds weight to previous research emphasising the importance of healthcare interventions to go beyond the biomedical and adopt a more patient-centred approach [17, 18]. Participants highlighted *the challenge of navigating the health system*, experiencing *a shift in what I expected from surgery*, and a strong desire to be acknowledged as *the expert in my own knee*. MacKichan et al. (2015) have previously evaluated barriers to optimal management of chronic pain after knee replacement from the perspective of healthcare professionals [19]. They identified the importance of managing patient expectations from surgery, and the complexity of referral pathways for chronic pain. Our study has shown that these are also priorities for patients. Mackichan et al. (2015) also highlighted that healthcare professionals in the NHS reported an absence of clear access points into care for people with post-surgical pain [19]. Our findings suggest that this has not improved in the decade since.

Our finding of participants feeling they had to *soldier on*, and the struggle of juggling multiple health priorities resonates with the themes identified by Moore and Gooberman-Hill (2020) who undertook qualitative interviews to explore reasons why some people with chronic pain after knee replacement do not seek help [20]. They identified a feeling among patients that they had to *‘get on with it’*, and a feeling that the responses of health-care professionals were discordant with their own experience of on-going pain. Our findings also resonate with Moore et al. (2015) who explored the impact on patients of undergoing revision hip surgery for deep prosthetic joint infection [13]. They identified frustration among patients that their concerns about symptoms were not heeded by clinicians. They also identified the ‘all-encompassing’ nature of experiencing a problematic joint replacement, and the difficulty of living with uncertainty and concerns about the future.

Participant’s narratives suggest that their experience and decision-making related to seeking care for their problematic knee replacement are influenced by moral discourses about being ‘*a good patien*t’, ‘*not wanting to cause trouble’* and protecting the NHS. Previous studies have highlighted that patients are highly sensitive to demands on the NHS and are anxious not to be seen as ‘timewasters’ or that they are using more than their share of health professionals time [21–23].

To our knowledge, this is the first qualitative study to specifically explore patients’ experiences of having a problematic knee replacement and the impact of undergoing revision knee joint replacement for aseptic, elective reasons. Qualitative research such as this study provides unique insight to patients’ experiences. This insight enables clinicians to reflect on their practice, to consider how the identified themes resonate with them and their view of patient experiences, and to identify steps they can take at an individual level to improve the experience for patients.

Our study has several limitations. Interviews were conducted via telephone. Telephone interviews reduce visual feedback, compared to face-to-face interviews, meaning the interviewer may lose important contextual information and thus the possibility to pursue an important issue. However, previous research has found that telephone interviews have several advantages over face-to-face, including increased access to geographically diverse subjects and allowing participants to remain on “their own turf”, leading to decreased social pressure, and increased rapport [24]. A limitation of thematic analysis lies in its interpretative nature, where the identification and analysis of themes is dependent on the researcher’s perspective. This subjectivity can lead to variations in the analysis, where different individuals are likely to identify different themes within the same dataset. A limitation of this study is that a purposive sample of participants was recruited from a Major Revision Centre within the NHS. Participants may have had more complex pathologies than those managed in less specialist units, resulting in more complex navigation of the healthcare system. Our participants included two persons from racial and ethnic minority groups, and we only explored the experience of living with a problematic knee replacement and undergoing revision surgery among people who were able to speak English at a sufficient level to take part in an in-depth interview. However, this may reflect the population receiving revision surgery as it is known that racial and ethnic minority groups are underrepresented in receiving primary knee replacement surgery [25].

The challenge and frustrations of navigating the healthcare system, and the need for patients to be recognised as the expert in their own knee, resonate with findings in the report of Baroness Cumberlege’s (2020) Independent Medicines and Medical Devices Safety Review [26]. In this review she described the current healthcare system in the UK (including the NHS) as “disjointed, siloed and unresponsive”. Her recommendations following the report aim to “build a system that listens, hears and acts”. While implementing system-wide changes will take significant time, listening, hearing, and acting should guide all clinicians in any interaction with patients. Doing so need not take more time but does require a change in approach. Communicating clearly, facilitating open and honest discussions, ensuring patients feel confident to ask questions and speak, and validating patients’ concerns will improve patient experiences. It is important that such open and clear communication is adopted throughout the clinical pathway, including during non-operative interventions, and the initial discussion of, and decision to proceed with, joint replacement. Several of our participants expressed that they wished they had been part of more open discussions about the risks, potential outcomes and alternatives to undertaking knee replacement surgery.

Currently in the UK neither NICE nor the NHS provide guidelines for long-term follow-up and monitoring of people with knee replacements and recommended clinical pathways for management of those with problematic knee replacement do not exist. Our findings also highlight the need for defined pathways, and specific interventions to support patients with problematic knee replacement. Our findings highlight the need for defined pathways, and specific interventions to support patients with problematic knee replacement. Specialist clinics and treatment programmes such as the STAR (Support and Treatment After joint Replacement) care pathway show promise for improving patient access to review and ongoing rehabilitation following primary joint replacement [11]. Consideration needs to be given to expanding such pathways to include patients who have undergone revision surgery and extending follow-up and support long-term. Future qualitative research should examine the experiences of specific patient groups, including younger patients, those still working, those with multiple comorbidities, patients from a range of racial and ethnic groups, and among those who do not speak English, to build an inclusive picture of patient experiences [27]. Doing so will enable future interventions to be developed that will support all patients who experience a problematic knee replacement in the best way possible.

Living with a problematic knee replacement and undergoing knee revision surgery has a significant impact on all aspects of patients’ lives. Our findings highlight the need for patients with problematic knee replacements to be supported to access care and assessment, and for long-term psychological and rehabilitation support before and after revision surgery.

### Electronic supplementary material

Below is the link to the electronic supplementary material.

**Additional File 1** Qualitative Interview Guide

**Additional File 2** Themes Subthemes and Example Codes

## Data Availability

Request for access to study data should be directed to the corresponding author (PN).

## Abbreviations

GP: General Practitioner
IMD: Index of Multiple Deprivation
MRI: Magnetic Resonance Imaging
NHS: National Health Service
UK: United Kingdom
