# Supplementary material for: The experience of patients undergoing aseptic, elective revision knee joint replacement surgery: a qualitative study
Source: BMC Musculoskelet Disord. 2024 Aug 29;25:676. doi: 10.1186/s12891-024-07778-3 (PMC11360607; doi:10.1186/s12891-024-07778-3)
Supplement: Supplementary file 1 — Additional File 1 Qualitative Interview Guide [file 12891_2024_7778_MOESM1_ESM.docx]

# ADDITIONAL FILE 1 Qualitative Interview Guide

*(Note that these questions are to be used as a guideline only)*

Thank you very much for speaking to me about your experience of living with knee pain and having surgery. I would like you to talk about anything that you think is important and has had an impact on your experience of living with a knee problem after the initial joint replacement surgery.

- Please can you tell me about when your knee problem started after the initial surgery?
- Can you talk to me about the impact that this knee problem had on you over time and now (family/work/social/feelings)?
- What did you do when you realised that you had a problem after the initial surgery?
- What help/care/treatment have you had?
- Can you tell me about your experience of healthcare/healthcare professionals?
- Can you talk me through the surgeries that you have had?
- Can you talk me through how you decided to have revision knee surgery?
- What were your hopes/expectations for your knee before having revision surgery? How do you feel about your knee now, 12 months on?
- What was it like in hospital and at hospital appointments?
- What advice would you give to other people (patients and professionals)
