# Supplementary material for: The experience of patients undergoing aseptic, elective revision knee joint replacement surgery: a qualitative study
Source: BMC Musculoskelet Disord. 2024 Aug 29;25:676. doi: 10.1186/s12891-024-07778-3 (PMC11360607; doi:10.1186/s12891-024-07778-3)
Supplement: Supplementary file 2 — Additional File 2 Themes Subthemes and Example Codes [file 12891_2024_7778_MOESM2_ESM.pdf]

ADDITIONAL FILE 2 Themes, Subthemes and Example Codes

Theme	Subtheme/s	Example codes
Soldiering on	Juggling hierarchy of health and social needs	No choice<br>Medication side effects<br>Prioritising<br>Multiple health conditions<br>Own health / others health
The challenge of navigating the health system		Frustration<br>Repeated visits<br>Ignored<br>Conflicting opinions<br>Lack of clarity<br>Anger<br>Multiple investigations
I am the expert in my own knee	Being a 'good' patient	I know my body<br>Trust in surgeon<br>Balance of power<br>Protecting the NHS<br>Others vs self<br>'Not one to make a fuss'
Shift in what I expected from surgery	It could have been much worse	Primary surgery hopes<br>Pain relief<br>'Some relief'<br>Reflection<br>Gratitude for any improvement
I am not the person I used to be	Me and my identity<br>My external identity	Disappointment in own body<br>Changed life expectations<br>Comparison to others<br>Relationship strain<br>Social exclusion
Lingering uncertainty		Shaken confidence<br>Ongoing 'threat'<br>No escape<br>Want to 'move on'<br>Unable to 'move on'<br>Reluctant acceptance
